# Supplementary material for: MetaRibo-Seq measures translation in microbiomes
Source: Nat Commun. 2020 Jun 29;11:3268. doi: 10.1038/s41467-020-17081-z (PMC7324362; doi:10.1038/s41467-020-17081-z)
Supplement: Supplementary file 10 — Supplementary Data 7 [file 41467_2020_17081_MOESM10_ESM.zip › File2/Confidence_High_Taxonomy/83681_out.krona.html]

Javascript must be enabled to view this page.

members
magnitude
magnitudeUnassigned
count
unassigned
taxon
rank

83681\_out

50

superkingdom
50
2

50
976
phylum

class
200643
50

50
171549
order

815
50
family


SRS012849\_contig\_number\_34763SRS015264\_contig\_number\_14460SRS020869\_contig\_number\_36886SRS045826\_contig\_number\_contig-100\_4194.117678SRS050299\_contig\_number\_4246SRS063985\_contig\_number\_contig-100\_4167.145473SRS105153\_contig\_number\_6536SRS144135\_contig\_number\_34091SRS146812\_contig\_number\_41820SRS148721\_contig\_number\_52038SRS150029\_contig\_number\_contig-100\_4822.96469
816
11
genus
50

species

SRS142712\_contig\_number\_5451
1
457395


SRS018049\_contig\_number\_201SRS018541\_contig\_number\_8798SRS045195\_contig\_number\_3402SRS052078\_contig\_number\_177SRS054590\_contig\_number\_1547SRS058070\_contig\_number\_6590SRS098655\_contig\_number\_3952SRS101268\_contig\_number\_37SRS1041145\_contig\_number\_39464SRS1055034\_contig\_number\_6311SRS148874\_contig\_number\_229SRS149879\_contig\_number\_14843
357276
12
species

species
2
1262744

SRS077024\_contig\_number\_934SRS149784\_contig\_number\_23689

species

SRS018984\_contig\_number\_10021
1
162156

671267
7

SRS016381\_contig\_number\_2483SRS018133\_contig\_number\_1257SRS024435\_contig\_number\_39186SRS048870\_contig\_number\_20354SRS058770\_contig\_number\_658SRS1041032\_contig\_number\_16487SRS1041136\_contig\_number\_23617
species

1
820

SRS024663\_contig\_number\_8004
species

species

SRS013098\_contig\_number\_38965SRS013800\_contig\_number\_12433SRS022071\_contig\_number\_35938SRS023346\_contig\_number\_3303SRS148511\_contig\_number\_14967SRS893230\_contig\_number\_contig-100\_2360.67006
6
821

species

SRS012273\_contig\_number\_37649SRS015960\_contig\_number\_contig-100\_3612.123726SRS018575\_contig\_number\_4766SRS048060\_contig\_number\_contig-100\_2558.77260SRS056259\_contig\_number\_13534SRS056519\_contig\_number\_12096SRS104636\_contig\_number\_17261
2292943
7

species
818
1

SRS013215\_contig\_number\_42

species
371601
1

SRS075341\_contig\_number\_18206
